# A pilot project harnessing surveillance systems to support clinicians providing clinical care for people diagnosed with hepatitis C in Victoria, Australia, September 2021 to 31 March 2022

**DOI:** 10.2807/1560-7917.ES.2024.29.29.2400028

**Published:** 2024-07-18

**Authors:** Mielle Abbott, Jennifer H MacLachlan, Nicole Romero, Nicole Matthews, Nasra Higgins, Alvin Lee, Mark Stoove, Tafireyi Marukutira, Brendan Quinn, Nicole L Allard, Benjamin C Cowie

**Affiliations:** 1 WHO Collaborating Centre for Viral Hepatitis at The Doherty Institute, Melbourne, Australia; 2 Victorian Government Department of Health, Melbourne, Australia; 3 Department of Infectious Diseases, University of Melbourne, Melbourne, Australia; 4 Public Health, Burnet Institute, Melbourne, Australia; 5 Australian Centre for the Prevention of Cervical Cancer, Melbourne, Australia; 6 School of Public Health and Preventive Medicine, Monash University, Melbourne, Australia; 7 Australian Research Centre in Sex, Health and Society, La Trobe University, Melbourne, Australia; 8 cohealth, Melbourne, Australia; 9 Victorian Infectious Diseases Service, Royal Melbourne Hospital, Melbourne, Australia; * These authors contributed equally to this work and share first authorship.; ** These authors contributed equally to this work and share last authorship.

**Keywords:** Hepatitis C, Surveillance, Cascade of Care, Department of Health, Australia

## Abstract

**Background:**

Active follow-up of chronic hepatitis C notifications to promote linkage to care is a promising strategy to support elimination.

**Aim:**

This pilot study in Victoria, Australia, explored if the Department of Health could follow-up on hepatitis C cases through their diagnosing clinicians, to assess and support linkage to care and complete data missing from the notification.

**Methods:**

For notifications received between 1 September 2021 and 31 March 2022 of unspecified hepatitis C cases (i.e. acquired > 24 months ago or of unknown duration), contact with diagnosing clinicians was attempted. Data were collected on risk exposures, clinical and demographic characteristics and follow-up care (i.e. HCV RNA test; referral or ascertainment of previous negative testing or treatment history). Reasons for unsuccessful doctor contact and gaps in care provision were investigated. Advice to clinicians on care and resources for clinical support were given on demand.

**Results:**

Of 513 cases where information was sought, this was able to be obtained for 356 (69.4%). Reasons for unsuccessful contact included incomplete contact details or difficulties getting in touch across three attempts, particularly for hospital diagnoses. Among the 356 cases, 307 (86.2%) had received follow-up care. Patient-management resources were requested by 100 of 286 contacted diagnosing clinicians.

**Conclusions:**

Most doctors successfully contacted had provided follow-up care. Missing contact information and the time taken to reach clinicians significantly impeded the feasibility of the intervention. Enhancing system automation, such as integration of laboratory results, could improve completeness of notifications and support further linkage to care where needed.

**Key public health message**

**What did you want to address in this study and why?**

Australia has committed to eliminating the public health threat posed by hepatitis C virus (HCV) infection by 2030. Follow-up of chronic hepatitis C notifications can potentially support elimination by assisting clinicians to provide the recommended care for their patients. Here, we wanted to understand if the Victorian Department of Health could follow-up on notifications via supporting the diagnosing clinicians to link patients to care and treatment.

**What have we learnt from this study?**

While missing clinicians’ contact details, notably in hospitals, was a challenge, 356 cases over 7 months in 2021–2022 could be assessed and 307 patients (> 85%) had received follow-up care i.e. clinicians ordered HCV RNA testing, offered treatment, or referred the patient. Many clinicians requested support. Clinicians identified patients’ unstable housing, difficulties getting to appointments, and competing health priorities as barriers to care.

**What are the implications of your findings for public health?**

Following up notifications with clinicians was feasible, however, incomplete clinician contact information was a barrier. Automatic data processes and reporting of all HCV RNA results can potentially support hepatitis C elimination by allowing public health officers to direct follow-up care to patients who most need it. Direct contact with patients should be considered. Piloting projects for follow-up of care after diagnoses in hospitals is important.

## Introduction

Hepatitis C is a leading cause of liver cancer and cirrhosis in Australia [1,2]. Liver cancer survival following diagnosis is low and it is a priority cancer in the National Cancer Roadmap [3]. Highly effective direct-acting antiviral (DAA) treatment reduces the risk of liver damage and liver cancer, and this was made available in Australia in 2016 [4]. Despite this, gaps in uptake of diagnostic testing and treatment remain [2,5,6]. Hepatitis C disproportionately affects people who have ever injected drugs, Aboriginal and Torres Strait Islander people, and people born in high hepatitis C prevalence countries [7], all of whom may face barriers to healthcare [8-10]. There is a global commitment to elimination of hepatitis C as a public health concern, which will require substantial progress in improving access to diagnosis, follow-up testing, and treatment [11].

Notification of a new diagnosis of hepatitis C is required from both laboratories and diagnosing clinicians in Australia. The preferred method of notification is via an electronic form, however, some clinicians choose to use paper forms. The notification requirement applies to any positive hepatitis C virus antibody (anti-HCV) test result, as well as to any positive ribonucleic acid (HCV RNA) test result [12]. While the anti-HCV confirms past exposure to hepatitis C, a positive RNA-HCV test result indicates an ongoing infection with HCV, and treatment is recommended. Although RNA testing is recommended for all anti-HCV positive individuals [13], gaps remain in the coverage of this follow-up testing [14]. Due to the requirement for an antibody positive test to reimburse an RNA test [15], the majority of cases are notified due to an anti-HCV test result. Notifications are classified using the surveillance definition as either newly acquired, where there is laboratory or clinical evidence that infection was acquired in the past 24 months [16]; or unspecified, where there is evidence that infection was acquired more than 24 months ago, or the duration is unknown (usually representing chronic infection) [17]. Most hepatitis C notifications in Australia are classified as unspecified [2]. Laboratory notifications include simple demographics such as the name, date of birth, sex, and place of residence of the individual notified, but no information on the context of diagnosis or risk factors relating to infection, which limits their utility in clinical and public health responses. This information, which can be provided by diagnosing clinicians, has however historically been poorly reported in Victoria.

Identifying people with hepatitis C who have not yet received HCV RNA testing to ascertain the presence of active infection requiring treatment is a priority for supporting elimination; it helps provide information on chronic infection prevalence, as well as on gaps in the HCV cascade of care, and can be used to support follow-up diagnostic testing and access to curative treatment. A study of those diagnosed in Victoria, Australia during 2001–2012, before the availability of DAAs, showed that only 41% had received follow-up HCV RNA testing [18]. However, this information has also historically not been available from surveillance systems; while compliance with laboratory notification requirements for anti-HCV is high [7,19], the results of HCV RNA testing (both detected and not detected) subsequent to positive anti-HCV tests have not been routinely reported.

In Australia, all permanent residents living with hepatitis C are eligible under Australia’s national universal health scheme (Medicare) to receive government-subsidised DAAs, including for re-infection [20]. General practitioners (GPs) and nurse practitioners (NPs) can prescribe DAAs for patients if experienced in care or in consultation with a non-GP specialist [20], and in Victoria, GP prescribing is increasing [5]. Victoria provides specialised support for community access to hepatitis C treatment through the Victorian Integrated Hepatitis C Nurses Network (IHCN) [21]. Barriers to GP-initiated treatment have included understanding how to confirm active infection using RNA testing and then treat hepatitis C [22]. A systematic response to hepatitis C notifications supporting initiation of follow-up care and treatment has been identified in Australia and globally, as a strategic priority supporting the elimination of hepatitis C as a public health concern [23,24].

The Coordinated Hepatitis response to Enhance the Cascade of Care by optimising existing Surveillance systems (CHECCS) pilot was funded by the Eliminate Hepatitis C Partnership (EC Australia) alongside other projects aimed at accelerating progress to HCV elimination. CHECCS was established in April 2021 [25] as a collaboration between researchers and the Victorian Department of Health (DH). The aims of the CHECCS pilot were (i) to assess feasibility of providing support to diagnosing clinicians to increase follow-up testing and treatment; and (ii) to improve collection of demographic and risk factor surveillance data with relevance to hepatitis C to guide the public health response. Here we describe the implementation of the CHECCS pilot and report on the project outcomes, including the impact of COVID-19 on the pilot.

## Methods

### Participants and study period

Notifications of hepatitis C unspecified cases (see Introduction for definition) were identified using Victoria’s Public Health Event Surveillance System (PHESS). The project included new notifications received between 1 September 2021 and 31 March 2022, which reflected a period of considerable impact from COVID-19 in Victoria [26].

Cases were excluded if they were aged < 18 years as they were not eligible for DAA treatment at the time [4]. Notifications from migration screening were excluded due to the frequent lack of clinician contact details. Notifications from blood donor screening, research study participants, and newly acquired cases were excluded as specific follow-up mechanisms were already in place for these notifications [27].

### Intervention

The project team worked closely with DH to ensure CHECCS was embedded within routine surveillance processes. Notifications eligible for inclusion in CHECCS were followed-up by a Public Health Officer (PHO) who worked 3 full days per week within the DH, contacting diagnosing clinicians by phone using details provided in laboratory notifications. Laboratories were contacted to ascertain complete diagnosing clinician contact details if information contained in the notification was insufficient. Initial follow-up occurred from 4 weeks after receipt of the notification. A maximum of three attempts were made to establish initial contact with the diagnosing clinician.

If at the first successful contact the diagnosing clinician was still undertaking clinical follow-up or identified the case was at risk of being lost to care, a second call was scheduled for 8 weeks after notification. This second call attempted to ascertain complete clinical data after clinical follow-up had been completed and assess if the initial contact had led to engagement or re-engagement in care.

Clinical advice regarding guideline-based care for hepatitis C and additional resources (including local referral pathways if available, clinician resources to guide testing and treatment, and patient resources) were offered to clinicians during the call and the clinicians’ reported need for these was recorded. Not all eligible cases were able to be followed up, due to staffing constraints exacerbated by the COVID-19 pandemic. A prioritisation framework for follow-up was established at project initiation [25]. Cases were deprioritised where contact with diagnosing clinicians was time-intensive and difficult (e.g. hospitals), or where it could be identified that cases were engaged with high caseload primary care clinics which typically have dedicated hepatitis C GP specialists.

Demographic, risk exposure and clinical data were collected at the time of the call via a structured form, including: Aboriginal and/or Torres Strait Islander status and country of birth; case history of injecting drug use status; case history of work in healthcare; whether the diagnosing clinician had ordered an HCV RNA test, and result if available; whether the diagnosing clinician had offered or prescribed treatment, or provided referral for treatment; reason for not providing testing or treatment.

A free text option was also available to capture any additional information.

These data were entered directly into PHESS. To help determine the feasibility of CHECCS, additional de-identified data were recorded by the PHO in a spreadsheet and stored in a secure data environment within DH, including: qualitative data on barriers to care and treatment for cases where the diagnosing clinician did not provide treatment; number of calls to laboratories to ascertain missing doctor details; whether a diagnosing clinician was sent resources; time taken by the PHO for phone calls to clinicians for the period 1 January 2022 to 31 March 2022 (after time constraints of the PHO were identified as a potential limitation to the follow-up of all notifying clinicians).

If preferred, clinicians could provide the above information via a project surveillance form by fax or email. When clinician contact was not possible, the PHO collected information from laboratory reports or hospital discharge summaries where available.

Data regarding care provision for people diagnosed in correctional facilities were obtained directly from the state-wide hepatitis service serving correctional facilities, through secure file sharing, overseen by DH and the Department of Justice and Community Safety. Resources and support were not offered for these clinicians given the dedicated state-wide hepatitis C clinical service delivered in correctional settings.

### Data analysis

De-identified data for hepatitis C notifications were extracted from PHESS and analysed using Microsoft Excel and Stata 14.2. Baseline characteristics of eligible cases during the project period were described, including age, sex (male, female, not stated), region of residence (metropolitan Melbourne or Regional Victoria based on Australian Bureau of Statistics classification [28]), Indigenous status, country of birth, history of injecting drug use (never, < 2 years ago, or ≥ 2 years ago), and healthcare worker status. Additionally, setting of diagnosis was described, and reported as community (general practice clinics, sexual health services, and Aboriginal Community Controlled Health Organisations), hospital (inpatient and outpatient), or correctional facilities.

Clinician contact-process information was also extracted, including presence of sufficient doctor details for contact; whether doctor contact was attempted and made; and if a second call was required at the 8-week time point (see Intervention).

For notified cases (anti-HCV positive) where information was provided by the diagnosing clinician (Figure), cascade of care indicators were described. These included the number and proportion of people who: received follow-up HCV RNA testing; were referred to another service; had no HCV RNA follow-up testing provided after the notification, but had had a previous negative RNA test result or prior successful treatment.

**Figure:**
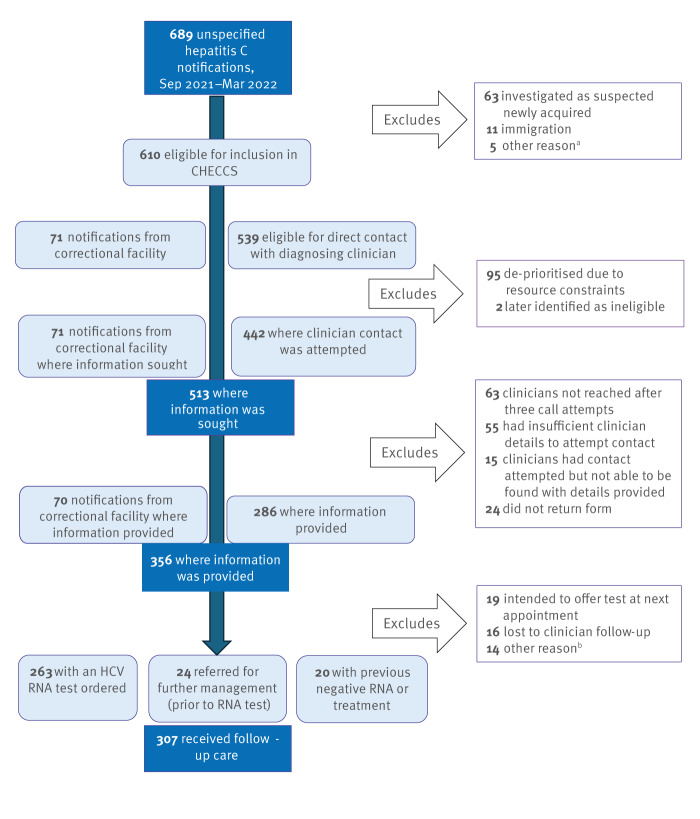
Flowchart of inclusion criteria, contact attempts with clinician, and care provision for hepatitis C cases, Victoria, Australia, 1 September 2021−31 March 2022 (n = 689 cases)

RNA: ribonucleic acid.

^a^ Includes those diagnosed via blood donor screening and those aged < 18 years.

^b^ Includes case refusal, case moving away, case records not able to be located by clinician.

‘Follow-up care’ represented by provision of an HCV RNA test; referral for further assessment; and/or ascertainment of previous negative testing or treatment history. Although cases with prior treatment or negative testing may still require ongoing HCV RNA monitoring due to the presence of risk factors for acquisition, as per Australian clinical guidelines, assessing the provision of this was not within the scope of the pilot.

These three indicators were also grouped together into a ‘case received follow-up care’ metric for analysis.

The proportion of those with an HCV RNA test result reported who were positive was assessed, as was the proportion of those HCV RNA positive who were offered antiviral treatment.

The ‘case received follow-up care’ outcome metric was analysed according to demographic factors including patient sex, age above or below the median, region of residence, setting, Indigenous status, country of birth, and injecting drug use status.

## Results

### Cases summary and baseline characteristics

A total of 689 cases of unspecified hepatitis C were notified between 1 September 2021 and 31 March 2022. Of these, 610 were identified as eligible for enhanced follow-up via the CHECCS project with the diagnosing clinician (Figure). Of the 610 cases, 411 (67.4%) were male; the median age was 48 years (interquartile range: 37–59 years; Table 1). A total of 392 cases (64.3%) resided in metropolitan Melbourne compared with 75.4% of the total population [29]. Most cases (337 cases, 55.3%) were diagnosed in community settings, with the remainder from hospitals (177 cases, 29.0%) and correctional facilities (71 cases, 11.6%).

**Table 1:** Demographic and clinical characteristics of hepatitis C cases eligible for follow-up, Victoria, Australia, 1 September 2021−31 March 2022 (n = 610 cases)

Case characteristic	Number of eligible cases	Proportion of total eligible cases (n = 610)
**Sex**		
Male	411	67.4%
Female	196	32.1%
Not stated	3	0.49%
**Age**		
Median age (IQR) in years	48 (37–59) -	
**Region of residence**		
Metropolitan Melbourne	392	64.3%
Regional Victoria	189	31.0%
Not stated	29	4.8%
**Diagnostic setting**		
Community	337	55.3%
Hospital	177	29.0%
Correctional facility	71	11.6%
Not stated	25	4.1%
**Indigenous status**		
Aboriginal and/or Torres Strait Islander	36	5.9%
Non-Indigenous	269	44.1%
Not stated	305	50.0%
**Region of birth**		
Australia	112	18.4%
Overseas	70	11.5%
Not stated	428	70.2%
**Injecting drug use history**		
Injecting drug use ≤ 2 years ago	52	8.5%
Injecting drug use > 2 years ago	57	9.3%
No injecting drug use history	74	12.1%
Not stated	427	70.0%
**Ever worked as healthcare worker**		
Yes	7	1.2%
No	78	12.8%
Not stated	525	86.1%

IQR: interquartile range.

‘Not stated’ includes cases where the diagnosing clinician was unaware of, or unable to, access relevant information, and those cases where the diagnosing clinician was not able to be contacted.

Despite attempts to gather further information by contacting the diagnosing doctor, information characterising notified cases was often incomplete, due partly to the number who were not able to be contacted (Figure). Indigenous status was missing for 50% of notifications. Moreover, region of birth and injecting drug use history were respectively lacking for 70% of cases, while healthcare worker status was not available for 86% of cases. Among all cases, 5.9% were identified as Aboriginal and/or Torres Strait Islander people, 11.5% were born overseas, 17.8% had any history of injecting drug use and 1.2% had ever worked as a healthcare worker (Table 1). Of the cases where sufficient clinician information was available to assess (401 cases; excluding hospital notifications without a specific clinician named), most clinicians (337, 84.0%) notified one case only. The highest number of cases notified by a single clinician was five, which occurred for two clinicians.

### Contact with clinicians and information collection

For 95 cases (15.6% of total eligible) no attempted contact was made due to de-prioritisation (see Methods), while two cases (0.3%) were only identified as ineligible after follow-up was attempted.

Among the remaining 513 cases where contact with the diagnosing clinician was attempted, contact was successful for 356 (69.4%); this was 37.9% for cases diagnosed in hospital and 79.4% in community settings (Table 2). The most common reasons for unsuccessful contact were not reaching the diagnosing clinician after three call attempts (63 of 513 cases, 12.3%), and insufficient details from laboratory notification to allow identification of the diagnosing clinician (55 of 513 cases, 10.7%; Figure). This included leaving a message with clinic reception staff for call back, finding out the best time to call the clinician back, sending an email to the clinician if provided, scheduling call backs and liaising with the reception on several occasions to leave a message. Information was available from nearly all correctional facility cases (98.6%, Table 2), due to the use of secure file transfer which was less impacted by these barriers.

**Table 2:** Diagnosing clinician contact and reported care uptake according to case characteristics among hepatitis C cases eligible for follow up, Victoria, Australia, 1 September 2021−31 March 2022 (n = 610 cases)

Case characteristics	Total eligible cases (N = 610)	Number of cases where follow-up care information was sought (N = 513)	Proportion of cases where follow-up care information was sought among total cases	Number of cases where follow-up care information was available (N = 356)	Proportion of cases where follow-up care information was available among total where it was sought	Number of cases who had follow-up care^a^ reported (N = 307)	Proportion of cases where follow-up care was reported among cases where information was available
**Sex**							
Male	411	347	84.4%	242	69.7%	209	86.4%
Female	196	163	83.2%	113	69.3%	97	85.8%
Not stated	NA	NA	NA	NA	NA	NA	NA
**Age^b^**							
< 48 years	305	268	87.9%	203	75.7%	175	86.2%
≥ 48 years	305	245	80.3%	153	62.4%	132	86.3%
Not stated	NA	NA	NA	NA	NA	NA	NA
**Region of residence**							
Metropolitan Melbourne	392	336	85.7%	229	68.1%	197	86.0%
Regional Victoria	189	159	84.1%	116	73.0%	100	86.2%
Not stated	29	NA	NA	NA	NA	NA	NA
**Diagnostic setting**							
Community	337	301	89.3%	239	79.4%	209	87.4%
Hospital	177	116	65.5%	44	37.9%	34	77.3%
Correctional facility	71	71	100%	70	98.6%	63	90.0%
Not stated	25	NA	NA	NA	NA	NA	NA
**Indigenous status**							
Aboriginal and/or Torres Strait Islander	36	35	97.2%	33	94.3%	32	97.0%
Non-indigenous	269	260	96.7%	254	97.7%	223	87.8%
Not stated	305	218	71.5%	69	31.7%	52	75.4%
**Country of birth**							
Australia	112	109	97.3%	105	96.3%	90	85.7%
Overseas	70	66	94.3%	62	93.9%	55	88.7%
Not stated	428	338	79.0%	189	55.9%	162	85.7%
**Injecting drug use history**							
Injecting drug use ≤ 2 years ago	52	49	94.2%	46	93.9%	34	73.9%
Injecting drug use > 2 years ago	57	56	98.2%	56	100%	52	92.9%
No injecting drug use history	74	71	95.9%	71	100%	67	94.4%
Not stated	427	337	78.9%	183	54.3%	154	84.2%
**Total**	**610**	**513**	**84.1%**	**356**	**69.4%**	**307**	**86.2%**

NA: not applicable, as data were suppressed to prevent re-identification and/or where the numbers of cases were too small for the percentages to be meaningful.

‘Not stated’ includes cases where the diagnosing clinician was unaware of or unable to access relevant information, and those cases where the diagnosing clinician could not be contacted. Totals other than the ‘total eligible case column’ will not add for sex, age, region of residence and diagnostic setting due to missing information and the suppression of uptake for these subgroups due to low numbers.

^a^ ‘Care’ represented by provision of an HCV RNA test; referral for further assessment; and/or ascertainment of previous negative testing or treatment history.

^b^ Age assessed as above or below the median; see Methods.

Doctor details were missing or incomplete in 129 cases initially, requiring a request of such details from testing laboratories; details were able to be ascertained in 74 cases, leaving 55 with no details available (Figure, 55 cases had insufficient clinician details and were excluded after information was sought).

### Call time resources

Of the 22 CHECCS calls to laboratories during the period assessed (for diagnosing clinician details or further results), duration averaged 4.1 min, while for the 73 calls to diagnosing doctors, duration averaged 5 min. There was an average of 1.7 call attempts before contact with the diagnosing doctor was successful. The average total time spent leaving messages was 4.9 min per case, of which ca 4.7 min was spent on hold and being transferred.

### Support for clinicians and re-engagement in care

One hundred clinicians (35.0% of the 286 contacted) requested resources to assist in hepatitis C management. Forty-seven clinicians who initially reported their patient was at risk of being lost to care received an additional follow-up call; 28 of the 47 reported contact with the CHECCS programme had led to engagement or re-engagement of their patient into care, including referral to the Victorian Integrated Hepatitis C Nursing service, or recall after being previously lost to follow-up.

### Care uptake

Of the 356 cases where information was able to be obtained through the diagnosing clinician, from laboratory reports or hospital discharge summaries, or via information transfer from correctional facilities, 263 (73.9%) had a follow-up HCV RNA test ordered by the diagnosing clinician at the time of diagnosis (Figure). A further 24 (6.7%) reported referring the patient to specialist care before HCV RNA testing, and 20 (5.6%) reported the patient had a record of treatment or a past negative HCV RNA test.

Of the 263 cases where a follow-up RNA test was ordered, information on the result was available for 237 cases, with 117 having positive HCV RNA (49.4%). Of those, 45 (38.5%) had treatment provided directly by the diagnosing clinician while 50 (42.7%) were referred for non-GP specialist care, 11 (9.4%) reported intending to offer treatment at the next appointment, and 11 (9.4%) reported not intending to provide treatment.

Loss to clinical follow-up was reported by the diagnosing doctor for 16 (4.5%) of the 356 cases with information available, and in another 19 (5.3%) cases the diagnosing clinician reported they intended to order the test at the next visit.

Overall, follow-up care (reported HCV RNA testing, referral, or prior treatment; see Methods) was reported as being provided for 307 (86.2%) cases (Figure, Table 2). This was lower for cases diagnosed in hospitals (77.3%) than those diagnosed in community settings (87.4%) or correctional facilities (90.0%). There was no evidence of disparity in follow-up care by age, sex, or area of residence. Further assessment of treatment provision or analysis by other demographic or clinical factors was limited by low case numbers (Table 1). While similarly limited by low data completeness, follow-up appeared to be less common in those with a recent history of injecting drug use (73.9%) than those with historical (92.9%) or no (94.4%) injecting drug use reported.

### Barriers to engagement in care

For cases where the diagnosing clinician did not provide care or treatment, qualitative data were collected from the clinician about barriers to engagement concerning 30 cases. There were multiple barriers to engagement in care identified by diagnosing clinicians including patient difficulty attending appointments, unstable housing, Medicare ineligibility for non-permanent residents, patients not feeling ready to have treatment for hepatitis C due to competing health priorities, not having access to a phone, and concerns about out-of-pocket expenses. Many GPs reported they would continue to have conversations with their patients to encourage treatment. The Victorian Integrated Hepatitis C Nursing service was commonly identified as an enabler to engaging with those cases where follow-up was challenging.

## Discussion

This paper describes the challenges and successes in implementing follow-up of hepatitis C notifications with diagnosing clinicians to promote linkage to care and treatment. Despite the pilot identifying high existing uptake of follow-up care after notification, one third of the clinicians contacted directly requested further resources. Over half of diagnosing clinicians caring for patients whom they identified as being at high risk of loss to follow-up reported that CHECCS supported engagement or re-engagement with hepatitis C care, often with the support of the IHCN.

The success of CHECCS in supporting clinicians and promoting engagement in HCV care needs to be balanced against the human resources required to follow-up notified positive anti-HCV results. Barriers to implementation identified included gaps in the availability of diagnosing clinician contact information, inability to contact clinicians when information was available as observed in other jurisdictions [30], resource constraints requiring the de-prioritisation of follow-up for a proportion of notified cases, and the considerable call time required to gather information. These gaps in completeness also limited robust assessment of metrics regarding successful contact of clinicians as an outcome of this intervention. This contrasted with high completeness for cases notified through correctional facilities where data were obtained through direct information transfer.

Gaps in laboratory data – particularly HCV RNA results – were a key barrier to the effective implementation of CHECCS. The automated notification of all HCV RNA results (including negative results) would greatly increase efficiency of follow-up by excluding individuals already known to be HCV RNA negative and therefore not requiring enrolment in treatment. Linkage to treatment data would further assist in identifying priority cases for follow-up. More complete reporting of clinician contact details from laboratories, and further integration of laboratory testing data with surveillance systems, would have provided for higher capture and efficiency. These additional data would also allow for fuller ascertainment of the cascade of care, allowing identification of any potential differential coverage among those with information not captured by CHECCS. At a systemic level, the implementation of reflex testing for all hepatitis C antibody positive results, which currently does not happen in Victoria, would provide assurance that all potentially positive hepatitis C RNA results have been captured, as all hepatitis C RNA positive individuals would be detected and therefore notified. This would also improve follow-up testing coverage and may support engagement in care, in addition to innovative strategies such as point of care and rapid testing approaches in priority settings [6].

The follow-up of hospital notifications was a particular challenge, with both completeness of diagnosing clinician details and successful contact through CHECCS lower than for community settings. Causes included frequent rotation of hospital junior medical staff and competing priorities exacerbated by additional pressure in the tertiary system due to COVID-19 during the pilot period [26]. Having a nominated clinician in the hospital – ideally affiliated with a hepatitis clinic or outreach service – who could follow-up and link those diagnosed to care would be an important service improvement to the provision of person-centred care. The implementation of direct contact with cases, rather than relying on diagnosing clinicians, could further address this gap and allow for enhanced linkage to care as required. Any approach of this type should be co-designed with consumers to ensure sensitivity to issues of stigma and discrimination [31].

When information could be gathered, there was evidence of high uptake of follow-up care, suggesting substantial progress compared with the pre-DAA era in Victoria [18]. Improvement in HCV RNA testing and increased engagement in care has been observed in Tasmania during 2020−2021 in response to notification follow-up with diagnosing clinicians, however, no significant increase in treatment uptake was found [32]. Nevertheless, evidence of variation in linkage to care across settings (particularly lower uptake in hospital settings) could provide guidance for potential prioritisation of those most at risk. Although robust assessment was not possible due to overlapping causes, there were insights into common reasons for lack of engagement in care and treatment, which can help guide future interventions, including a review of ineligibility for subsidised treatment via Medicare. Those diagnosed in correctional facilities had a higher uptake of follow-up care through a dedicated treatment programme in a controlled environment.

Evidence from Europe has indicated that dedicated follow-up of those lost to care may be effective in improving RNA testing uptake and treatment outcomes [33,34], however, further robust evidence is required regarding impact. There is also scope for the improvement of routinely collected surveillance data completeness in the European setting, particularly regarding risk factors for infection and cultural and linguistic diversity [35], which could be supported by the type of follow-up approach piloted in Victoria.

Due to resource constraints for the pilot, follow-up regarding care provision information ceased if the diagnosing clinician reported that the case had been referred to another service for care. This may be a remaining gap given referral cannot guarantee that the case was seen, or ultimately provided with care (including HCV RNA testing for those referred prior). Increasing data capture (for example via data linkage) would give a fuller picture of the uptake of follow-up care for newly diagnosed cases of hepatitis C in Victoria, and this work has been initiated since the completion of the CHECCS pilot.

For the first time in Victoria, CHECCS captured key data points regarding care and treatment uptake for hepatitis C through routine surveillance. If this information were able to be collected in an ongoing way, this would allow rapid assessment of the current status of the hepatitis C cascade of care for newly diagnosed cases and allow for real-time intervention to optimise linkage to care for all Victorians diagnosed with hepatitis C.

## Conclusion

This pilot provides evidence for the feasibility and effectiveness of the use of surveillance systems to enhance access to care and treatment for hepatitis C. With refinement of processes and expansion of data capture, follow-up of notified cases could be a key tool in pursuing hepatitis C elimination.
